# Spontaneous regression of quiescent gestational trophoblastic disease after pregnancy: a case report

**DOI:** 10.1186/s12905-019-0794-2

**Published:** 2019-07-23

**Authors:** Yoshiyuki Okada, Shingo Miyamoto, Takashi Mimura, Tetsuya Ishikawa, Akihiko Sekizawa, Koji Matsumoto

**Affiliations:** Department of Obstetrics and Gynecology, Showa University School of Medicine, 1-5-8 Hatanodai, Shinagawa-ku, Tokyo, 142-8666 Japan

**Keywords:** Quiescent gestational trophoblastic disease, Pregnancy, Spontaneous regression

## Abstract

**Background:**

A persistent low-level elevation of serum human chorionic gonadotropin (hCG) without clinical or radiological evidence of pregnancy or tumors was recently defined as quiescent gestational trophoblastic disease (Q-GTD). Whether patients with Q-GTD should be treated or allowed to become pregnant remains unclear. We herein report a rare case of Q-GTD in which the hCG level spontaneously returned to normal after a successful pregnancy.

**Case presentation:**

The patient was a 37-year-old primigravida who presented with a persistent low-level elevation of hCG after uterine evacuation of a hydatidiform mole. There was no evidence of neoplasia in the uterus or distant metastasis. The low-level elevation of hCG persisted for at least 2 years but never exceeded 200 mIU/mL. The patient had a successful pregnancy at the age of 40 years.

**Conclusions:**

Interestingly, her hCG level subsequently normalized without chemotherapy. The present case may imply the safety and therapeutic effect of pregnancy in women with Q-GTD.

## Background

In recent decades, quiescent gestational trophoblastic disease (Q-GTD) has been defined as an inactive or benign form of GTD without detectable lesions that is diagnosed by a persistent low-level elevation of the serum human chorionic gonadotropin (hCG) level, usually in the range of 50 to 100 mIU/mL and typically < 200 mIU/mL, for ≥3 consecutive months [1, 2]. Serum hCG is not detected in normal women. For diagnosis of Q-GTD, a urinary hCG test and oral contraceptive pills are useful to exclude false-positive hCG results (phantom hCG) and pituitary hCG elevation, respectively [3]. In women with false-positive hCG, urine hCG test results are negative because heterophile antibodies to hCG are not excreted in the urine due to their large size, whereas the production of pituitary hCG can be inhibited with oral contraceptive pills. It is postulated that the low-level elevation of hCG may result from the presence of fully differentiated syncytiotrophoblasts, which produce a small amount of hCG. In most patients with Q-GTD, the serum hCG concentration returns to normal within 12 months [4]. In previous studies, therefore, close surveillance without chemotherapy has been recommended for Q-GTD until malignant disease is detected [2, 5]. However, 10 to 25% of Q-GTD reportedly progresses to malignant disease [1, 6]. In addition, little is known about the safety of pregnancy in reproductive-age women with long-term quiescent hCG. Moreover, whether patients with Q-GTD should be treated or allowed to become pregnant remains unknown.

We herein report a rare case of Q-GTD in which the hCG level spontaneously returned to normal after a successful pregnancy. This case may provide insight into the mechanism of spontaneous hCG normalization in patients with Q-GTD.

## Case presentation

A 37-year-old primigravida was referred to our hospital because of a diagnosis of a hydatidiform mole at 10 weeks of gestation. She had no family history of GTD. Her serum hCG level was 35,000 mIU/ml, and transvaginal ultrasound demonstrated an abnormal mass of 65 × 38 mm with a specific “snow-storm” pattern in the uterine cavity. The uterus was evacuated immediately, and the pathological diagnosis of the removed specimens was a complete hydatidiform mole. Although a second curettage procedure was performed at 11 weeks of gestation, no residual molar tissue was found.

**Fig. 1:**
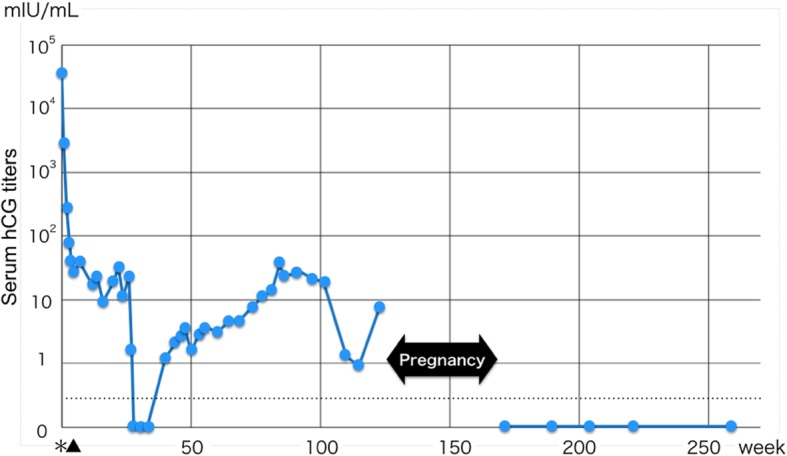
Persistent low-level elevation of serum hCG after uterine evacuation of a hydatidiform mole. The low-level elevation of the hCG titer persisted for at least 2 years but became undetectable after pregnancy. The dotted line shows the cutoff value. The asterisk shows the first evacuation, and the triangle shows the second evacuation

The serum hCG level decreased to within the normal range temporarily after molar evacuation, but it gradually increased again at 40 weeks after evacuation (Fig. 1). Computed tomography, magnetic resonance imaging, and hysteroscopy revealed no tumor. The serum hCG level persisted in the range of 5 to 50 mIU/ml. False-positive hCG (i.e., “phantom hCG”) was excluded by a urine hCG test. Oral contraceptive pills had no effect on the hCG titer. These evaluations led to a diagnosis of Q-GTD.

The patient decided to avoid chemotherapy after a discussion with the gynecologic oncologist. At the age of 40 years, she wanted to have a child. After 2 years of observation of a low hCG level, we advised that she attempt pregnancy. She was conceived naturally and had an uneventful and successful delivery. The placenta appeared macroscopically normal. Her hCG level returned to normal 2 months after delivery (Fig. 1). At the time of this writing (5 years post-delivery), she was clinically well with negative hCG.

## Discussion and conclusions

We have herein reported a rare case of Q-GTD in which the hCG level spontaneously returned to normal after a successful pregnancy. To the best of our knowledge, this is the first report of spontaneous Q-GTD regression following a successful pregnancy in Japan; two similar cases of Q-GTD have been reported in the UK and US [4, 7].

Agarwal et al. [4] analyzed the clinical data of 76 patients with persistently elevated but declining hCG levels 6 months after evacuation of hydatidiform moles. In their report, one woman aged 39 years exhibited hCG normalization after pregnancy, although detailed data were not shown. In another report, the hCG level in a 34-year-old woman with Q-GTD spontaneously returned to normal after two pregnancies [7]. Women with Q-GTD are usually required to avoid pregnancy because the high hCG level during pregnancy confuses the clinical picture [5]. However, these observations suggest that pregnancy may be permitted for childbearing women with Q-GTD after a certain period of hCG observation. Furthermore, pregnancy may contribute to spontaneous hCG normalization in women with Q-GTD, although the mechanism is unknown. One may speculate that a small fraction of syncytiotrophoblasts producing small amounts of hCG in the uterus may come out together with the placenta at the time of delivery. In previous studies, however, hysterectomy did not reduce the titers of circulating hCG, indicating the presence of hCG-secreting syncytiotrophoblasts outside the uterus [8]. Accordingly, a highly elevated hCG level during pregnancy might contribute to subsequent hCG normalization in women with Q-GTD by a yet unknown mechanism.

The present case, together with two cases reported in previous publications, may imply the safety and therapeutic effect of pregnancy in women with long-term quiescent hCG. However, this finding will need to be confirmed by a large-scale, multicenter retrospective survey of Q-GTD cases.

Interestingly, her hCG level subsequently normalized without chemotherapy. The present case may imply the safety and therapeutic effect of pregnancy in women with Q-GTD.

## Data Availability

All data generated or analyzed during this study are included in this published article and its supplementary information file.

## Abbreviations

hCG: Human chorionic gonadotropin
Q-GTD: Quiescent gestational trophoblastic disease
